# Expectations for Mendelian randomization research in *PLOS One*

**DOI:** 10.1371/journal.pone.0337199

**Published:** 2025-11-18

**Authors:** Johanna Pruller, James Mockridge, George Vousden

**Affiliations:** Public Library of Science (PLOS), San Francisco, California, United States of America; PLOS: Public Library of Science, UNITED KINGDOM OF GREAT BRITAIN AND NORTHERN IRELAND

## Growth in problematic Mendelian randomization research

Mendelian randomization (MR) can be used to establish a causal effect of a modifiable exposure (e.g., smoking) and specific outcomes (e.g., lung cancer) using genetic variations as a proxy for the modifiable factors of interest. The method is described in more detail in Williams *et al*. [1]. Over the last few years, there has been a significant increase in the number of MR studies being published, with a wide variation in research quality [1].

**Fig 1:**
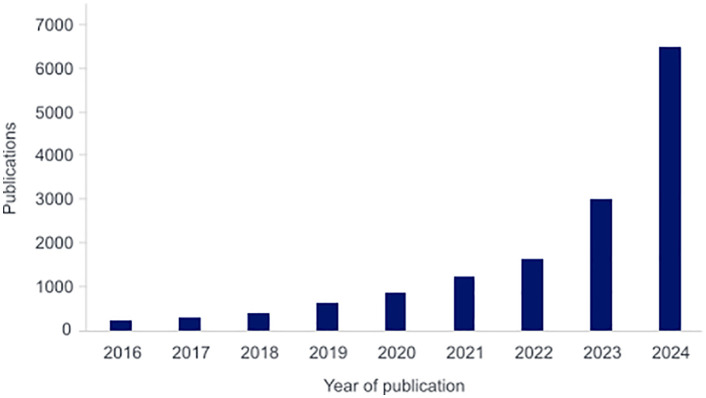
Number of publications with “Mendelian randomization” or “Mendelian randomisation” in the title or abstract. **Data were obtained from** Dimensions.ai (accessed September 30th 2025).

Since 2022, the publication of MR studies has almost doubled each year, with just over 6500 studies published in 2024 alone (Fig 1). Publication numbers remain high, with over 5000 articles already published in 2025. *PLOS One* has received nearly 1800 submissions of MR studies in 2024 alone, and 2025 shows a similar trend with 919 manuscripts submitted by August 2025.

This Editorial highlights the policies and processes that *PLOS One* has put in place to ensure sufficient quality of the studies we publish and outlines our expectations for MR studies. We will continue to publish MR studies that have been conducted rigorously and contribute to the base of academic knowledge, as required by our publication criteria [2].

## Requirements to publish Mendelian randomization research in *PLOS One*

At *PLOS One*, every submission, regardless of study type, is screened against specific criteria before an Academic Editor is invited to handle the manuscript. The specific nature of this screen, which is performed by editorial staff, depends on the article type and content, and 40% of all submissions are rejected at this stage for non-compliance with PLOS policies for research reporting [3] and ethical publishing practices [4].

After concerns around the quality of MR studies were raised by members of our community, we introduced a specific pre-screening for adherence of MR studies to our publication criteria in April 2024. These checks require that authors supply a completed STROBE-MR checklist [5]. Editorial staff evaluate the information requested in the checklist against our criteria requiring that analyses are performed to a high technical standard, with non-compliant manuscripts being rejected. Requesting the STROBE-MR checklist also ensures that the prospective Academic Editor is provided with all the necessary information they require to evaluate the submission.

A significant number of MR studies submitted to *PLOS One* address research questions that have been considered in previous publications, occasionally even using identical methodology. Examples include the relationship between female reproductive factors and breast cancer or the effect of smoking on laryngeal cancer. At *PLOS One,* we require submitted research to contribute to the academic literature and clearly reference and discuss similar work [2]. As such, we would expect authors to clearly articulate the contribution their submission makes to the academic literature. This is assessed as part of the initial editorial pre-screening, and manuscripts that do not sufficiently justify the research question and the contribution the research makes are rejected.

MR studies can be used to investigate certain associations, especially for research questions where clinical trials are impossible. However, it is essential that authors conduct MR studies so that the chosen methods are robust and support the conclusions drawn. This includes carefully considering the suitability of the dataset to address the chosen research question, and a careful selection of appropriate statistical analysis tools. It is also important to consider that a strictly *in silico* MR analysis, without further *in vitro* or *in vivo* validation, cannot definitively prove causality. To ensure robustness of the conclusions, a critical discussion of the findings in light of recent literature is essential, as is the validation of findings in, at minimum, a second independent dataset. At initial submission, editorial staff assess whether the findings have been validated in this manner and reject those that do not meet this criterion.

**Fig 2:**
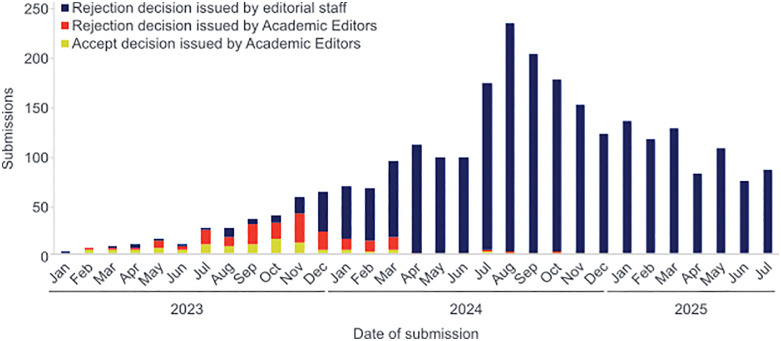
Outcomes of submissions to *PLOS One* using Mendelian randomization (MR) methodology from January 2023 to July 2025 (date of submission). Numbers of monthly submissions using MR methodology that were (a) rejected by Academic Editors (red), (b) rejected by editorial staff (blue), or (c) accepted by Academic Editors (yellow). Since the additional editorial screening step was introduced in April 2024, the share of rejections issued by editorial staff has increased, and acceptances have decreased.

## Preventing publication of poor-quality MR research

Our editorial screening process assesses MR submissions against our expectations detailed above and has led to a high number of submissions being rejected. The triage process, performed by editorial staff, has significantly reduced the number of submissions that enter peer review, which in turn allows our expert editorial board members and the subject-specific reviewers to focus their efforts on good-quality MR study submissions. This process has also ensured all manuscripts reporting the results of MR studies meet our minimal standards as well as community expectations for this study design.

This editorial screening has impacted the number of rejection decisions that are issued by *PLOS One* Academic Editors and editorial staff. Before December 2023, when editorial staff began developing the screening policy based on established standards in the community, the vast majority of rejection decisions were issued by members of the editorial board. By April 2024, when the triage process was in place, close to 100% of rejection decisions were issued by editorial staff (Fig 2). We are grateful that, with the input of our Academic Editors, we were able to develop a policy that has been highly successful in *PLOS One* retaining only submissions that describe research performed to a high technical standard that adhere to appropriate reporting guidelines and community standards.

*PLOS One* evaluates research on the basis of scientific validity, strong methodology, and high ethical standards, selecting research that contributes to academic knowledge. We accept a wide range of submissions from primary research to protocols and systematic reviews. As such, we still welcome submissions of high-quality MR studies, and are honored that so many authors choose to publish their research in *PLOS One*.
